# Open Drug Scenes Survey in Brazilian cities: main findings from São Paulo, Fortaleza, and Brasília

**DOI:** 10.1590/1980-549720250008

**Published:** 2025-03-03

**Authors:** Clarice Sandi Madruga, Kátia Isicawa de Sousa Barreto, Danilo Seabra, André Constantino Miguel, Cláudio Jerônimo da Silva, Gleuda Simone Apolinário, Guilherme Godoy, Lidiane Nogueira Rebouças, Natália Alexandre Ferreira, Quirino Cordeiro, Rogério Adriano Bosso, Ronaldo Ramos Laranjeira

**Affiliations:** I Universidade Federal de São Paulo – São Paulo (SP), Brazil.; II Associação Paulista para Desenvolvimento da Medicina – São Paulo (SP), Brazil.; III Washington State University – Spokane (WA), United States of America.; IV Casa Civil, Office of the Deputy Governor of the State of São Paulo – São Paulo (SP), Brazil.; V Secretaria da Proteção Social – Fortaleza (CE), Brazil.; VI Universidade de São Paulo – Ribeirão Preto (SP), Brazil.

**Keywords:** Ill-housed persons, Crack cocaine, Social vulnerability, Mental health services, Unified health system

## Abstract

**Objective:**

The latest edition of the Open Drug Scenes Survey in Brazilian Cities (LECUCA) investigated social vulnerability, health, and the use of the Psychosocial Care Network by attendees of open drug scenes (ODSs) involving crack cocaine in São Paulo, Fortaleza, and Brasília between 2021/2022.

**Methods:**

Since 2016, LECUCA has used Time-Location Sampling (TLS) to select probabilistic samples representative of the population of ODS attendees.

**Results:**

We interviewed 579 participants in São Paulo, Fortaleza, and Brasília, obtaining a response rate of 75%. We found no difference in ODS attendees regarding the prevalence of sociodemographic indicators and time living in the ODS. The prevalence values of attendees who had never been homeless before living in the ODS and those living in their homes were equally high in the three capitals. Fortaleza stood out for having lower rates of homelessness and limited access to specialized health services, whereas Brasília had high rates of searching for emergency services due to drug use and greater access to all modalities of health and assistance services. Unprotected sex was prevalent over one third of ODSs attendees, and none of the capitals had more than half of the attendees testing for tuberculosis and sexually transmitted infections. Rates of pregnancy complications were high in all three capitals, with São Paulo accounting for the lowest rates.

**Conclusion:**

LECUCA provides significant subsidies to governmental and institutional managers, aiming at catalyzing the formulation of public policies and care strategies based on data and evidence.

## INTRODUCTION

Worldwide, one in 17 people used at least one type of drug in 2022. According to the United Nations Office on Drugs and Crime (UNODC) this rate has continued to grow, having increased by 23% in the last 10 years^1^. Different psychoactive substances lead to different types of harm to the public and individual health. While the use of opiates (natural or synthetic, licit or illicit) plays a key role in the scenario of illicit drug use in most of the world, in Latin America cocaine (aspirated or smoked) is the most consumed illicit drug, after marijuana^1^, and the one that most results in search for treatments^2,3^. Crack cocaine consumption is on the rise in Brazil, where inequality constantly permeates and enhances the process of social exclusion intrinsic to severe substance use disorders^4-6^.

The use of drugs in public places is a social phenomenon common to any urban center. However, the context in which individuals, of all ages and socioeconomic profiles, cluster to obtain and use drugs among their peers is only referred to as Open Drug Scene (ODS) when the drug in question is illegal. In the case of Brazil, ODSs are associated with the combination of the distribution and consumption of crack cocaine with an even more prominent aspect, which is the social vulnerability faced by its attendees, who are mostly homeless^7-9^. Even though it is a common phenomenon in several locations worldwide, there are few scientific publications whose authors characterize and map this phenomenon^9-14^.

Although the first ODS in Brazil was acknowledged in the city of São Paulo, with the well-known "Cracolândia" (a São Paulo region where individuals gather to buy and consume crack cocaine openly; *Cracolândia* could be freely translated into "Crackland")^15-17^, similar phenomena occur in greater or lesser proportions in several Brazilian cities^18^. The manifestation of this urban phenomenon is inherently variable, as it arises from the intersection of social vulnerability and substance use disorders. This phenomenon frequently leads to conflicts with other social groups or players within the city^19,20^.

The profile of the attendees of the largest ODS in São Paulo has been systematically monitored by the Open Drug Scenes Survey in Brazilian Cities (*Levantamento de Cenas de Uso em Capitais* – LECUCA) through a probabilistic epidemiological survey on historical series since 2016, which uses specific sampling methods to obtain representative samples^21^. The results of this survey have been paramount for improving initiatives and services in the areas of health care, social assistance, public security, housing, work, and income; in addition, its relevance in the context of São Paulo has led to its replication in open drug scenes of capitals in the Northeast and Midwest of Brazil.

In the present study, we aim to understand the profile of ODSs attendees in São Paulo, Fortaleza, and Brasília as well as provide reflections on the particularities of each territory. Understanding the needs of these populations contributes to the development of more contextualized and effective approaches and to inspire evidence-based policies and practices for open drug scenes throughout the national territory.

## METHODS

In this study, ODS was defined as the place where at least 15 still (not moving between two locations) substance users are verified for at least three consecutive days. Data were collected between June 2021 and March 2022 in São Paulo; in August 2021, in Fortaleza; and between April and May 2022, in Brasília.

### Exploratory study

Exploratory excursions were carried out to define the ODS of each capital and delimit its geographical perimeters. The ODS of the Luz neighborhood region, in São Paulo (known as "Cracolândia"), included eight perimeters; in Fortaleza, in the Moura Brasil neighborhood (known as "Oitão Preto"), four perimeters were delimited; and in Brasília, in the Centro Comercial Sul region (known as "Buraco do Rato"), five perimeters were delimited. Each perimeter was mapped with basic georeferencing and the start and end points of the scans were alternated so that each interviewer traveled different routes during the approaches.

### Sampling

The Time-Location Sampling (TLS) method is an extension of the Location-Based Sampling method, a probabilistic sampling method used to study rare populations gathering at specific locations^21^. The selection method is based on selecting the sample from the target population at randomly determined times at specific locations. This method has been used for assessing regulars in nightclubs and for investigating populations at high risk of developing sexually transmitted infections^22^. TLS provides for predetermined site visits at randomized time periods^23^. Time was considered as a Primary Sampling Unit (PSU), which was randomized into two levels: days and times. The sampling also has a randomization per location resource, which is the starting point of the scan. Considering the high population density of some of the territories being studied (especially in São Paulo), the simple scanning protocol of the predefined perimeters was adopted. Twenty scanning cycles in every ODS perimeter were performed in each capital, where interviewers crossed each ODS perimeter addressing all eligible attendees on days and times previously randomized.

### Sample

To calculate the sample size of the ODS in São Paulo, a finite population of about 1,608 people was considered (LECUCA 2019 count). The classic sampling formula with 95% confidence (Z=1.96) and sampling error of up to 5% for 10% estimates was used, resulting in a sample of approximately 122 individuals. In Fortaleza and Brasília, without previous population data, the exploratory study estimated up to 500 attendees in each city — which, with the same parameters, led to a sample of 98 individuals per capital. Considering the high probability of refusals, all LECUCA editions adopt the full scan of the perimeter, inviting all individuals who meet the inclusion criteria for the interview. Thus, all attendees of legal age present in the ODS perimeters were invited to the interview, with the exception of:

- those who were using crack cocaine at the moment of the interviewers’ approach;
- those displaying distress or extreme behavioral agitation;
- unconscious attendees.

### Refusals and nonresponses

The sampling procedure undermines the determination of precise refusal rates, as the same individual may refuse to participate in one cycle and accept to do so in another. The repetition control was carried out by asking about previous participation in the initial approach. Refusals occurred in 25% of the initial approaches. Nonresponse was considered to be the abandonment of the interview with less than 20% of the questionnaire completed, resulting in the exclusion of less than 10% of the questionnaires. The final sample summed up a total of 573 participants, composed of: N=357 in São Paulo; N=140 in Fortaleza; and N=82 in Brasília.

### Instrument

he structured interviews (15 minutes of duration in average) were based on a multiple-choice questionnaire covering topics such as sociodemographic characteristics, physical and mental health, Sexually Transmitted Infections (STIs), social vulnerability, risk behaviors, reproductive health, substance use, history of healthcare services use, and motivation for treatment. The questionnaire included closed-ended questions for comparability between historical series in São Paulo and other capitals, adapted for each region through focus groups with professionals from the Psychosocial Care Network (*Rede de Atenção Psicossocial* – RAPS) at the ODS. New questions (added in São Paulo's 5th wave) were validated by cognitive interviews performed during a pilot study.

### Measures

#### Sociodemographic characteristics

The sociodemographic section of the questionnaire followed the standards established by the Brazilian Institute of Geography and Statistics (IBGE)^24^ and included questions on: gender (options: man, woman, transgender); age (numerical, open-ended question); race/skin color (options: white, black, Asian, Indigenous and "mixed-race" [originally called "*Pardo*," according to Brazil's self-reported racial classification official census and demographic surveys proposed by IBGE, which refers to individuals of mixed-race ancestry, often with a combination of European, African, and Indigenous heritage]). The sociodemographic investigation also included level of education (options: illiterate, elementary school, high school, and higher education, complete and incomplete); marital status (options: single, married/common-law marriage, divorced, widowed); paid activity (options: yes, no); governmental benefits (yes, no, and benefit type); social support network (options: yes, no); and types of support (multiple options: people and services in the ODS region, other services, family, friends).

#### Adaptations made to the instrument

The questionnaire was adapted given the constant changes in the ODS context since the survey's first edition in São Paulo as well as due to regional differences of the other two ODSs included in the latest edition. Adaptations were made in the topic related to the use of the health and assistance network, available at the ODS, including information related to the services offered to each context, including their references (usually addresses). The qualitative analysis of the cognitive interviews led to further changes in the substance use section, including "Merla", (a by-product of cocaine commonly used in Brasília), "Crack no Bombril" (use of steel wool for smoking crack cocaine), and "Balão de Maconha" (known as "blunts", Marijuana + Tobacco). The option "Leprosy" was also included in the checklist of physical conditions as cases were reported during the training with local healthcare teams in Fortaleza. Finally, the checklist of exposure to adverse events was also updated with the inclusion of an alternative to report history of experiencing labor analogous to slavery within social rehabilitation services’ settings.

#### Data processing and analysis

A data entry stage was necessary, as the ODS context presented additional risks for interviewers using computerized systems for data collection. The data imputation process included three levels of checks, followed by stages of cleaning, fitting, consistency verification, coding, and conversion to STATA SE13 software. Standard deviations stratified by capital were calculated without sample weighting. Due to the small sample size of transgender people, it was not possible to segment the total sample by gender. Pearson's χ^2^ hypothesis tests or Fisher's Exact Test were applied according to the nature and distribution of the data in each indicator to verify the homogeneity between the three subpopulations (capitals as independent samples).

### Ethical aspects

The LECUCA interview protocol was approved by the Research Ethics Committee of UNIFESP with opinions available on *Plataforma Brasil* (the national platform for managing the ethical review and approval process of research involving human beings in Brazil) under registration numbers: CAAE (Certificate of Presentation for Ethical Consideration) 46249121.7.0000.5505 and CAAE 46249121.7.3001.5553.

More information on LECUCA is available on the study website: https://lecuca.uniad.org.br/sobre-o-levantamento/sobre-o-lecuca/.

## RESULTS

### Sociodemographic profile

According to Table 1, we found no relevant differences regarding gender distribution in the three surveyed capitals, with a predominance of men with a mean age of 37.8 years in the three capitals (standard deviation [SD]=10.5; minimum age, 18 years; maximum age, 77 years). Regarding racial classification, most participants from all three ODSs self-reported to be mixed-race. The majority of participants were single and did not attend high school. The proportion of illiterate individuals was significantly higher in Fortaleza.

**Table 1:** Sociodemographic and vulnerability indicators.

Variable		SÃO PAULO (N=357)			FORTALEZA (N=140)			BRASÍLIA (N=82)			TOTAL (N=579)		
		n; %	95%CI		n; %	95%CI		n; %	95%CI		n; %	95%CI	
**Gender**													
	Man	262; 73.8	68.96	78.13	95; 68.8	60.57	76.06	58; 72.5	61.56	81.27	415; 72.3	68.61	75.94
	Woman	80; 22.5	18.47	27.19	39; 28.3	21.33	36.41	21; 26.3	17.67	37.12	140; 24.4	21.08	28.13
	Transgender	13; 3.7	2.13	6.21	4; 2.9	1.08	7.53	1; 1.3	0.17	8.58	18; 3.1	1.99	4.94
**Age**													
	18–25	43; 14.0	10.51	18.32	16; 11.6	7.19	18.16	9; 11.0	5.75	19.94	68; 12.9	10.28	16.02
	26–60	253; 82.1	77.44	86.04	121; 87.7	81.01	92.24	67; 81.7	71.67	88.75	441; 83.5	80.10	86.45
	60 or over	12; 3.9	2.22	6.75	1; 0.7	0.1	5.04	6; 7.3	3.28	15.51	19; 3.6	2.30	5.58
**Skin color** *													
	White	90; 25.9	21.52	30.74	13; 10.0	5.87	16.54	13; 15.9	9.36	25.59	116; 20.7	17.55	24.28
	Black	88; 25.3	20.98	30.14	46; 35.4	27.59	44.05	28; 34.2	24.6	45.18	162; 28.9	25.31	32.83
	Mixed-race	166; 47.7	42.48	52.97	69; 53.1	44.41	61.56	38; 46.3	35.73	57.3	273; 48.7	44.62	52.90
	Asian	2; 0.6	0.14	2.28	0.0			0.0			2; 0.4	0.09	1.42
	Indigenous	2; 0.6	0.14	2.28	2; 1.5	0.38	6.01	3; 3.7	1.16	10.91	7; 1.2	0.60	2.60
**Marital status**													
	Single	270; 75.8	71.11	80.02	94; 71.2	62.84	78.35	56; 69.1	58.12	78.33	420; 73.8	70.04	77.27
	Divorced/separated	33; 9.3	6.66	12.77	15; 11.4	6.94	18.07	8; 9.9	4.96	18.7	56; 9.8	7.65	12.58
	Married/common-law marriage	44; 12.4	9.32	16.22	20; 15.2	9.95	22.4	10; 12.4	6.71	21.62	74; 13.0	10.48	16.03
	Widowed	9; 2.5	1.32	4.8	3; 2.3	0.73	6.88	7; 8.6	4.13	17.2	19; 3.3	2.14	5.18
**Level of education** *													
	Illiterate	6; 1.7	0.76	3.71	10; 7.6	4.13	13.68	3; 3.7	1.16	10.91	19; 3.3	2.14	5.18
	Elementary school	232; 65.2	60.05	69.96	86; 65.6	57.05	73.33	46; 56.1	45.08	66.55	364; 64.0	59.93	67.82
	High school	103; 28.9	24.44	33.88	28; 21.4	15.13	29.30	25; 30.5	21.39	41.41	156; 27.4	23.90	31.24
	Technical education/college degree	15; 4.2	2.55	6.88	7; 5.3	2.55	10.85	8; 9.8	4.90	18.48	30; 5.3	3.71	7.45
**Current paid activity** *		5			1								
	No	228; 68.7	63.46	73.45	76; 58.9	50.16	67.14	57; 70.4	59.41	79.4	361; 66.6	62.52	70.46
**Source of income**					0								
	Has no income	188; 53.1	47.88	58.27	73; 56.2	47.44	64.5	42; 51.9	40.89	62.64	303; 53.6	49.49	57.72
	Social benefit	188; 43.2	38.13	48.45	50; 38.5	30.43	47.17	33; 40.7	30.48	51.88	236; 41.8	37.76	45.89
	Informal income	2; 0.6	0.14	2.24	2; 1.5	0.38	6.01	0.0			4; 0.7	0.27	1.87
	Retirement	5; 1.4	0.59	3.36	2; 1.5	0.38	6.01	4; 4.9	1.84	12.6	11; 1.9	1.08	3.49
	Other source	6; 1.7	0.76	3.73	3; 2.3	0.74	6.98	2; 2.5	0.61	9.52	11; 1.9	1.08	3.49
**Housing**					0								
	Stable housing	34; 9.5	6.90	13.08	67; 51.5	42.91	60.08	19; 23.5	15.39	34.05	120; 21.2	17.99	24.73
	Unstable housing	86; 24.2	19.98	28.89	19; 14.6	9.48	21.86	5; 6.2	2.56	14.15	110; 19.4	16.34	22.87
	Homelessness*	236; 66.3	61.20	71.03	44; 33.9	26.18	42.47	57; 70.4	59.41	79.4	337; 59.4	55.33	63.41
**How is the open drug scene frequented**													
	Live/sleep in the ODS most days	200; 56.8	51.57	61.92	69; 54.3	45.54	62.86	41; 58.6	46.56	69.64	310; 56.5	52.27	60.57
	Spend the day and sleep in the ODS sporadically	19; 5.4	3.46	8.32	8; 6.3	3.16	12.17	5; 7.1	2.96	16.26	32; 5.8	4.15	8.13
	Spend the day, but never sleep in the ODS	101; 28.7	24.19	33.66	26; 20.5	14.28	28.46	21; 30.0	20.30	41.90	148; 27.0	23.40	30.84
	Visit the ODS only to buy drugs	32; 9.1	6.49	12.59	24; 18.9	12.95	26.73	3; 4.3	1.36	12.69	59; 10.7	8.41	13.63
**Time in the open drug scene**					2								
	Less than a month	16; 4.6	2.8	7.3	2; 1.6	0.39	6.11	7; 9.1	4.34	18.05	25; 4.5	3.05	6.56
	One month to one year	55; 15.6	12.18	19.82	18; 14.1	9	21.3	13; 16.9	9.98	27.13	86; 15.4	12.67	18.69
	One to five years	79; 22.4	18.37	27.12	38; 29.7	22.36	38.24	16; 20.8	13.04	31.46	133; 23.9	20.51	27.60
	Five to ten years	64; 18.2	14.48	22.58	15; 11.7	7.15	18.61	12; 15.6	8.99	25.66	91; 16.3	13.49	19.65
	Ten years or over	138; 39.2	34.22	44.42	55; 43.0	34.61	51.75	29; 37.7	27.44	49.11	222; 39.9	35.86	43.99
**History of homelessness before attending the ODS** *		121; 35.1	30.20	40.28	92; 23.3	16.56	31.82	26; 35.6	25.36	47.38	175; 32.5	28.69	36.61
**Origin**													
	Treatment institution (Therapeutic Community/hospital)	10; 2.9	1.54	5.25	3; 3.2	1.02	9.56	1; 1.2	0.17	8.47	14; 2.7	1.59	4.47
	Shelter center (hostel/hotel)	21; 6.0	3.95	9.06	4; 4.3	1.59	10.92	4; 4.9	1.84	12.6	29; 5.5	3.87	7.86
	Other institution (inmate/youth detention center)	24; 6.9	4.64	10.07	3; 3.2	1.02	9.56	3; 3.7	1.18	11.03	30; 5.7	4.03	8.08
	Own home or family members’	255; 73.1	68.15	77.47	71; 75.5	65.72	83.25	49; 60.5	49.34	70.65	375; 71.6	67.54	75.27
**Has social support network**		132; 41.0	35.73	46.47	60; 50.0	41.06	58.94	9; 11.1	5.82	20.17	201; 38.4	34.35	42.69
	Social support source	190; 59.0	53.53	64.27	60; 50.0	41.06	58.94	61; 88.9	79.83	94.18	322; 61.6	57.31	65.65
	Services in the territory	37; 16.1	11.87	21.45	6; 7.5	3.37	15.88	11; 15.3	8.58	25.73	54; 14.1	10.98	18.02
	Other services	12; 5.2	2.98	8.99	3; 3.8	1.19	11.17	6; 8.3	3.74	17.54	21; 5.5	3.61	8.29
	Family	149; 64.8	58.36	70.71	52; 65.0	53.8	74.76	49; 68.1	56.28	77.91	250; 65.4	60.52	70.06
	Acquaintances/friends*	46; 20.0	15.3	25.7	34; 42.5	32.03	53.69	18; 25.0	16.23	36.45	98; 25.6	21.51	30.29

* Indicates significance (p<0.05) in the homogeneity test between the capitals (χ^2^ Test or Fisher's Exact Test, depending on the indicator analyzed).

### Vulnerability indicators and social support network

As shown in Table 1, the ODSs studied presented similar rates of attendees with no income, but they differ in terms of the proportion of attendees receiving benefits (higher prevalence in Brasília. While most attendees of ODSs in São Paulo and Brasília were homeless, we verified a higher proportion (51.5%) of attendees who reported having a permanent home in Fortaleza.

A high proportion of attendees reported having come from their homes to the ODS in all three capitals, and just over a third of respondents in São Paulo and Brasília reported having been homeless before attending the ODS.

The proportion of newcomers (up to one year in the ODS) was similar in the three capitals, with São Paulo presenting more individuals frequenting the ODS for five years or more. In all capitals, more than half of the interviewees live in the ODS (they spend most of the day and sleep there at night). The social support network available for the three samples varied: in São Paulo and Fortaleza, almost half had no one to rely on in cases of emergencies, whereas in Brasília this accounted for only 11.1% of individuals. Among those reporting social support, family was the most frequently reported source, followed by acquaintances and friends.

### Substance use and search for emergency services

According to Table 2, not all ODS attendees report using crack cocaine. This prevalence was not homogeneous among the capitals, with similar rates (almost 80%) in São Paulo and Fortaleza, but with less than a third of the participants referring to the use of crack cocaine in Brasília. Rates of snorted cocaine and marijuana were also lower in Brasília compared to the other ODSs studied. In contrast with the lower rates of consumption, Brasília also presented significant higher rates of individuals reporting the need for emergency services due to drug use, with a third of respondents referring to recently seeking emergency services.

**Table 2:** Health indicators.

VARIABLE		SÃO PAULO			FORTALEZA			BRASÍLIA			TOTAL (N=579)		
		n; (%)	95%CI		n; (%)	95%CI		n; (%)	95%CI		n; %	95%CI	
**Substance use (previous year)**													
	Crack cocaine*	277; 78.9	74.32	82.88	108; 77.7	69.96	83.9	25; 30.9	21.67	41.88	410; 71.8	67.96	75.35
	Aspirated cocaine*	133; 37.9	32.95	43.1	56; 40.3	32.4	48.71	10; 12.2	6.63	21.37	199; 34.8	30.99	38.80
	Marijuana	220; 62.2	56.96	67.07	99; 71.2	63.08	78.19	34; 41.5	31.2	52.53	353; 61.4	57.34	65.30
	Alcohol	226; 63.7	58.51	68.52	89; 64.0	55.65	71.63	63; 76.8	66.33	84.81	378; 65.6	61.64	69.40
	Inhalant solvent	30; 8.7	6.14	12.18	14; 10.1	6.02	16.38	10; 12.2	6.6	21.44	54; 9.5	7.38	12.26
**Use of emergency services due to consumption**													
	No	244; 73.5	68.46	77.98	89; 73.0	64.31	80.15	35; 48.6	37.15	60.22	368; 70.0	65.90	73.74
	Yes, in the last year	62; 18.7	14.83	23.25	12; 9.8	5.64	16.61	24; 33.3	23.3	45.15	98; 18.6	15.52	22.20
	Yes, over a year ago	26; 7.8	5.38	11.27	21; 17.2	11.45	25.05	13; 18.1	10.68	28.87	60; 11.4	8.95	14.43
**History of STIs and tuberculosis testing**													
	Tuberculosis	205; 61.4	56.02	66.47	70; 53.9	45.17	62.3	41; 50.0	39.19	60.81	316; 57.9	53.68	61.96
	HIV	198; 59.3	53.91	64.44	79; 61.2	52.49	69.32	49; 59.8	48.68	69.92	326; 59.8	55.63	63.86
	Syphilis*	202; 60.7	55.29	65.79	62; 47.7	39.18	56.34	47; 57.3	46.27	67.68	311; 57.1	52.86	61.17
	Hepatitis B*	146; 43.8	38.59	49.24	47; 36.2	28.3	44.83	45; 54.9	43.89	65.41	238; 43.7	39.55	47.88
	Hepatitis C*	145; 43.4	38.17	48.8	42; 32.3	24.77	40.89	43; 53.1	42.08	63.8	230; 42.2	38.11	46.40

* Indicates significance (p<0.05) in the homogeneity test between the capitals (χ^2^ Test or Fisher's Exact Test, depending on the indicator analyzed).

### History of STIs testing and reproductive health

Less than 60% of the studied population reports a history of having already tested for STIs (Table 2). Only Brasília had at least half of the ODS population tested for all STIs. On average, Fortaleza had the lowest testing rate, followed by São Paulo and Brasília. Tuberculosis and HIV were the most tested infections, while hepatitis B and C were the least tested, falling below 50% in São Paulo and Fortaleza.

Unprotected sex was reported by over a third of attendees of the ODSs (35% in the general sample), 31.9% in São Paulo, 46.4% in Fortaleza, and 28.6% in Brasília. The use of long-acting reversible contraceptives (implants or intrauterine devices) was reported only in São Paulo, with a prevalence of use in 16.2% of women. Most women reported having had at least one pregnancy (full-term or not). The average number of pregnancies in life was between 3.4 (São Paulo) and 5.0 (Fortaleza), and 3.5 in Brasília. The age of the first pregnancy was 18 years, on average, with a minimum age of 10 years and a maximum age of 36 years (the means by capitals are: São Paulo, 18.3 years; Fortaleza, 16.5 years; and Brasília, 19.3 years). Among women with a pregnancy history, about 70% reported a history of at least one complication in previous pregnancies. The most reported pregnancy complications were miscarriage (spontaneous), which reached 63.3% of cases in Fortaleza, and preterm birth, reaching 54.6% in Brasília.

### History of service network use in life

According to Table 3, the most used services were General Hospitals and Health Centers (*Unidades Básicas de Saúde* – UBS), with more than half of the studied population referring to a history of having accessed these facilities at least once in their lives. Greater access to the healthcare and assistance network stands out in Brasília, where the majority (85.4%) reported having accessed general hospitals and more than half of the sample attended CAPS-AD. The federal capital also had high rates of respondents who reported having accessed mutual aid groups such as Alcoholics Anonymous (AA). We also observed a significant difference between the capitals regarding the use of psychiatric hospitals and CAPS-AD, in which Fortaleza had much lower rates than the other capitals, with only 8.53% and 9.3% of service history in the two services, respectively.

**Table 3:** History of use of RAPS services (use in life).

	SÃO PAULO			FORTALEZA			BRASÍLIA			TOTAL		
	N; %	95%CI		N; %	95%CI		N; %	95%CI		N; %	95%CI	
General Hospitals*	247; 70.6	65.57	75.13	72; 55.8	47.07	64.21	70; 85.4	75.8	91.57	389; 69.4	65.39	73.03
Psychiatric hospitals*	72; 20.9	16.89	25.5	11; 8.5	4.76	14.82	17; 20.7	13.2	31.02	100; 18.0	15.00	21.41
UBS*	175; 50.6	45.3	55.84	89; 69.0	60.42	76.43	53; 64.6	53.58	74.32	317; 56.9	52.75	60.98
CAPS	44; 12.9	9.7	16.86	12; 9.3	5.33	15.74	6; 7.3	3.28	15.51	62; 11.2	8.83	14.13
CAPS-AD*	74; 21.6	17.53	26.26	34; 26.4	19.42	34.7	47; 57.3	46.27	67.68	155; 28.0	24.39	31.87
Community center*	57; 16.7	13.07	21.01	34; 26.4	19.42	34.7	34; 41.5	31.2	52.53	125; 22.6	19.30	26.29
Private service professional*	6; 1.7	0.71	3.86	5; 3.9	1.61	9.04	13; 15.8	9.36	25.59	24; 4.3	2.92	6.40
Alcoholics anonymous*	32; 9.4	6.69	12.95	6; 4.6	2.09	10.04	20; 24.4	16.2	34.98	58; 10.5	8.19	
Narcotics anonymous	50; 14.5	11.18	18.68	6; 4.6	2.09	10.04	15; 18.3	11.25	28.33	71; 12.8	10.26	15.85
Therapeutic communities*	55; 16.1	12.55	20.38	14; 10.8	6.5	17.57	28; 34.1	24.6	45.18	97; 17.5	14.59	20.95
Religious entities	49; 14.3	10.96	18.42	12; 9.3	5.33	15.74	10; 13.4	7.52	22.79	71; 12.8	10.29	15.90

* Indicates significance (p<0.05) in the homogeneity test between the capitals (χ^2^ Test or Fisher's Exact Test, depending on the indicator analyzed).

## DISCUSSION

The replication of the survey to the capitals Fortaleza and Brasília enabled the understanding of new perspectives on the phenomenon that involves the formation and consolidation of open crack cocaine scenes in Brazil. The results allowed us to understand the profiles of the attendees of the open drug scenes studied, in addition to reflecting on the particularities of each territory and common needs.

We observed a parallel between the three ODSs studied regarding the existence of a favorable context for people who use illicit substances to find an environment for peer consumption — in which, mostly, they end up living for a prolonged time. The high number of homeless people in São Paulo and Brasília is an indication of the way to relate to local residents, when compared to the low prevalence in Fortaleza. Unlike the other capitals, Fortaleza's ODS integrates with the local community in a different way. Authors of an ethnographic research in the region identified the presence of women selling drugs, showing that the sale of crack cocaine is incorporated into the "daily chores routine"^25^. Although it met the criteria that define LECUCA's ODS concept, the territory in Fortaleza was considered "hybrid" and characterized by the reclusive use of the drug.

In São Paulo, the ODS has unique characteristics in relation to the spatiality of the territory, the behavior, and the social identity of its attendees. One material aspect of identification with the ODS in São Paulo is the public use of pipes^26^, contrasting with the more reclusive use in Fortaleza and the discreet use in Brasília, where crack cocaine consumption may be underreported. Taken together, these observations lead to speculation about differences between capitals in terms of stigma related to drug use.

Our results offer insights into the complex relationship between homelessness and drug use: approximately two-thirds of the attendees had lived in their own homes or with family members before transitioning to life in the ODS. A small proportion of our samples from all three ODSs studied had experienced homelessness before attending the ODS, challenging the narratives that allude to the use of crack cocaine as a result of living on the streets. Based on our findings, substance use disorder often preceded homelessness, undermining economic stability and social connections. However, as noted in other studies^27^, crack consumption may also function as an adaptive response to the hardships experienced on the streets.

The high rates of unprotected sex and complications in previous pregnancies combined with the high rates of miscarriage in Fortaleza and Brasília indicate the need for family planning initiatives for highly vulnerable women who attend the open drug scenes. In São Paulo, these rates are lower due to the timid but existing offer of long-acting reversible contraceptives (implants or intrauterine devices).

The rates of emergency care due to drug use in Brasília also deserve special attention. The significantly higher rates found in the Brazilian capital may be attributed to the circulation of more potent drugs such as variants of "merla." However, this scenario is mitigated by greater access to healthcare services such as UBS and CAPS-AD.

The rates of access to network services of the Brazilian Unified Health System (SUS) and the Unified Social Assistance System (SUAS) show the importance of the existence of services in the vicinity of the ODS. With the highest levels of accessibility for virtually all the health and social assistance services investigated, the ODS in Brasília is characterized by having a CAPS-AD at its epicenter. The attendees of the ODS of São Paulo also benefit from the proximity to different health and social assistance services, with even higher rates of access to the Psychosocial Care Center for Adult Care (*CAPS* *Adulto*). This is evidenced by the high prevalence of use of the UBS in Fortaleza, which is the only SUS service available in the territory. With these findings we demonstrate the need for public policies that promote the implementation of effective primary and secondary prevention strategies, inside and outside the territories of the ODSs.

Furthermore, windows of opportunity for early intervention before individuals migrate to ODSs are missed, thus denouncing the urgent demand for more integrated approaches, from the primary to the specialized healthcare network, which do not have protocols for screening substance abuse from the point of view of secondary prevention and early intervention.

The diverse vulnerabilities of individuals living in ODSs require an integrated care network with effective intersectoral coordination. Only well-connected and synchronized services can address the complex needs of those in extreme vulnerability^28^.

It is also crucial that social and health networks provide care that is sensitive to gender diversity and the specific health needs of women, through qualified and tailored approaches. This aligns with the SUS principle of integrality, which emphasizes human rights and social factors, prioritizing a care trajectory focused on resocialization alongside the technical aspects of health.

Our study has limitations that impact the interpretation and generalization of the results. The main one concerns obtaining representative samples of only one ODS per capital, although there are other scenes in these cities. Although the ODSs chosen are considered the main ones by RAPS professionals and confirmed by the exploratory study, they do not reflect the entire population of attendees of these capitals, requiring caution in generalizing the data. Possible self-selection and nonresponse biases should also be considered. Moreover, the exclusion of attendees with a high degree of intoxication may have generated bias, as we excluded these severe cases from the sample. Although the questionnaire is adapted to this population, there is still potential response bias, which may affect reliability. However, we believe that the improvement of issues over previous editions in São Paulo and the exclusion of the most intoxicated individuals may mitigate this bias.

The Open Drug Scene Survey in Brazilian Cities (LECUCA) provides significant subsidies to governmental and institutional managers, aiming at catalyzing the formulation of public policies and care strategies grounded in data and evidence. Our findings highlight that all ODSs studied require more integrated, coordinated, and systematic actions. In addition to ensuring access to health and support services, these actions must include complementary services that enhance recovery opportunities for individuals with substance use disorders in open drug scenes.
