# Supplementary material for: Open Drug Scenes Survey in Brazilian cities: main findings from São Paulo, Fortaleza, and Brasília
Source: Rev Bras Epidemiol. 2025 Mar 3;28:e250008. doi: 10.1590/1980-549720250008 (PMC11884818; doi:10.1590/1980-549720250008)
Supplement: Translation

# Translation: Levantamento de Cenas de Uso de capitais: achados principais de São Paulo, Fortaleza e Brasília

**DOI:** 10.1590/1980-549720250008.2

**Authors:** Clarice Sandi Madruga, Kátia Isicawa de Sousa Barreto, Danilo Seabra, André Constantino Miguel, Cláudio Jerônimo da Silva, Gleuda Simone Apolinário, Guilherme Godoy, Lidiane Nogueira Rebouças, Natália Alexandre Ferreira, Quirino Cordeiro, Rogério Adriano Bosso, Ronaldo Ramos Laranjeira

**Affiliations:** I Universidade Federal de São Paulo – São Paulo (SP), Brasil.; II Associação Paulista para Desenvolvimento da Medicina – São Paulo (SP), Brasil.; III Washington State University – Spokane (WA), Estados Unidos da América.; IV Casa Civil, Gabinete do Vice-Governador do Estado de São Paulo – São Paulo (SP), Brasil.; V Secretaria da Proteção Social – Fortaleza (CE), Brasil.; VI Universidade de São Paulo – Ribeirão Preto (SP), Brasil.

**Keywords:** Pessoas mal alojadas, Cocaína Crack, Vulnerabilidade social, Serviços de saúde mental, Sistema Único de Saúde

## Abstract

**Objetivo:**

A última edição do Levantamento de Cenas de Uso de Capitais (LECUCA) investigou indicadores de vulnerabilidade social, de saúde e uso de serviços de saúde e assistência entre frequentadores das cenas abertas de uso (CAU) de crack em São Paulo, Fortaleza e Brasília entre 2021/2022.

**Métodos:**

Desde 2016, o LECUCA utiliza a amostragem por Tempo-Localização (TLS) para selecionar amostras probabilísticas representativas da população de frequentadores da CAU.

**Resultados:**

Foram entrevistados 579 participantes em São Paulo, Fortaleza e Brasília, com índice de resposta de 75%. Os frequentadores das CAUs não se diferenciam nas prevalências de indicadores sociodemográficos e tempo na cena. As prevalências de frequentadores que nunca estiveram em situação de rua antes de viver na CAU e provenientes de casa foram igualmente altas nas três capitais. Fortaleza se destaca com menores índices de situação de rua e de acesso a equipamentos de saúde especializados, enquanto Brasília apresenta índices elevados de busca por emergência devido ao consumo de drogas e maior acesso a todas as modalidades de serviço de saúde e assistência. Mais de um terço dos frequentadores das CAUs pratica sexo desprotegido e nenhuma das capitais teve mais da metade dos frequentadores testados para Tuberculose e Infecções Sexualmente Transmissíveis (ISTs). Os índices de problemas na gestação foram altos entre todas as capitais, com os menores índices em São Paulo.

**Conclusão:**

O LECUCA oferece subsídios importantes para gestores governamentais e institucionais, com o intuito de catalisar o desenvolvimento de políticas públicas e estratégias de atendimento fundamentadas em dados e evidências.

## INTRODUÇÃO

No mundo, uma a cada 17 pessoas usou pelo menos um tipo de droga em 2022. O Escritório das Nações Unidas para Crime e Drogas (UNODC) destaca que esse índice tem um crescimento contínuo, tendo aumentado 23% nos últimos 10 anos^1^. Diferentes substâncias psicoativas levam a diferentes prejuízos para a saúde pública e individual. Enquanto o uso de opiáceos (naturais ou sintéticos, lícitos ou ilícitos) ocupam um papel central no cenário de uso de drogas ilícitas em quase todo o mundo, na América Latina a cocaína (aspirada ou fumada) é a droga ilícita mais consumida, depois da maconha^1^, e a que mais gera busca por tratamentos^2,3^. O consumo de crack desponta no Brasil, onde a desigualdade atravessa e potencializa o processo de exclusão social intrínseco aos transtornos aditivos graves^4-6^.

O uso de drogas em lugares públicos é um fenômeno social comum a qualquer centro urbano. Todavia, o contexto em que indivíduos, de todas as idades e perfis socioeconômicos, se aglomeram para obter e usar uma droga entre pares só é referido como Cena Aberta de Uso (CAU) quando a droga em questão é ilegal. No caso do Brasil, as CAUs estão associadas à combinação da distribuição e do consumo de crack com um aspecto, talvez, ainda mais saliente, que é a vulnerabilidade social enfrentada por seus frequentadores, que se encontram, na sua maioria, em situação de rua^7-9^. Mesmo sendo um fenômeno comum em várias localidades do mundo, são poucas as publicações científicas que caracterizam e mapeiam esse fenômeno^9-14^.

Embora o reconhecimento da primeira CAU no Brasil tenha sido em São Paulo, com a conhecida "cracolândia"^15-17^, fenômenos semelhantes ocorrem em maiores ou menores proporções em diversas cidades brasileiras^18^. A variabilidade da manifestação desse fenômeno urbano é esperada, uma vez que combina a vulnerabilidade social com transtornos aditivos. Muitas vezes esse fenômeno ocorre de forma conflituosa com os demais atores sociais da cidade^19,20^.

O perfil dos frequentadores da maior CAU de São Paulo vem sendo monitorado sistematicamente pelo Levantamento de Cenas de Uso em Capitais (LECUCA) através de levantamento epidemiológico probabilístico de séries históricas desde 2016, o qual utiliza métodos de amostragem específicos para obter amostras representativas^21^. Os resultados desse levantamento têm sido fundamentais para o aprimoramento de ações e serviços nas áreas da saúde, assistência social, segurança pública, moradia, trabalho e renda, e sua relevância no contexto paulistano levou à sua replicação em cenas de uso das capitais no Nordeste e Centro-Oeste do país.

O estudo busca compreender o perfil dos frequentadores das CAUs em São Paulo, Fortaleza e Brasília, bem como fornecer reflexões sobre as particularidades de cada território. A compreensão das necessidades dessas populações colabora para o desenvolvimento de abordagens mais contextualizadas e eficazes, bem como para inspirar políticas e práticas baseadas em evidência para cenas de uso em todo território nacional.

## MÉTODOS

Neste estudo, CAU foi definida como o local em que são verificados no mínimo 15 usuários de substâncias parados (não em trânsito entre duas localidades), por pelo menos três dias consecutivos. A coleta de dados realizou-se entre junho de 2021 e março de 2022 em São Paulo; em agosto de 2021 em Fortaleza e entre abril e maio de 2022 em Brasília.

### Estudo exploratório

Foram realizadas excursões exploratórias para definir a CAU de cada capital e delimitar seus perímetros geográficos. A CAU da região da Luz, em São Paulo ("Cracolândia"), incluiu oito perímetros; em Fortaleza, no Bairro Moura Brasil ("Oitão Preto"), foram delimitados quatro; e em Brasília, no Centro Comercial Sul ("Buraco do Rato"), cinco. Cada perímetro foi mapeado com georreferenciamento simples, e os pontos de início e fim das varreduras foram alternados para que cada entrevistador percorresse diferentes rotas durante as abordagens.

### Amostragem

O método Tempo-Localização (*Time-Location Sampling*, ou TLS) é uma extensão do método da Amostragem Baseada no Local, um método de amostragem probabilística utilizado para estudar populações raras que congregam em locais específicos^21^. O método de seleção baseia-se em selecionar a amostra da população-alvo em momentos determinados aleatoriamente em localizações específicas. Tal método tem sido utilizado para a avaliação de frequentadores em clubes noturnos e para a investigação de populações com alto risco de contaminação de doenças sexualmente transmissíveis^22^. O TLS prevê visitas no local pré-determinado em períodos de tempo randomizados^23^. Considerou-se o tempo como Unidade Primária de Amostragem (*Primary Sampling Unit — PSU*), que foi randomizado em dois níveis: dias e horários. A amostragem também conta com uma instância de randomização por localização, sendo esse o ponto de início da varredura. Levando em consideração a alta densidade populacional de alguns territórios estudados (especialmente em São Paulo), adotou-se o protocolo de varredura simples dos perímetros pré-definidos. Vinte ciclos de varredura foram realizados em cada capital, onde os entrevistadores percorreram cada perímetro da CAU abordando todos os frequentadores elegíveis nos dias e horários previamente definidos.

### Amostra

Para calcular o tamanho da amostra da CAU em São Paulo, considerou-se uma população finita de cerca de 1.608 pessoas (contagem LECUCA de 2019). Utilizou-se a fórmula clássica de amostragem com 95% de confiança (Z=1,96) e erro amostral de até 5% para estimativas de 10%, resultando em uma amostra de aproximadamente 122 indivíduos. Em Fortaleza e Brasília, sem dados populacionais prévios, o estudo exploratório estimou até 500 frequentadores em cada cidade, o que, com os mesmos parâmetros, levou a uma amostra de 98 indivíduos por capital. Dada a alta probabilidade de recusas, todas as edições do LECUCA adotam a varredura completa do perímetro, convidando para entrevista todos os indivíduos que atendem aos critérios de inclusão. Assim, todos os frequentadores maiores de idade presentes nos perímetros da CAU foram convidados para a entrevista, com exceção de:

- aqueles que estavam usando crack no momento da abordagem;
- aqueles com intensa agitação comportamental;
- aqueles com sinais de intoxicação grave;
- frequentadores desacordados.

### Recusas e não respostas

A natureza da amostragem dificulta determinar índices precisos de recusa, já que um participante pode recusar em um ciclo e aceitar em outro. O controle de repetição foi feito perguntando sobre participação prévia na abordagem inicial. As recusas ocorreram em 25% das abordagens. Considerou-se como não resposta o abandono da entrevista com menos de 20% do questionário preenchido, resultando na exclusão de menos de 10% dos questionários. A amostra final, com 573 participantes que completaram entre 80% e 100% do questionário, foi composta por: N=357 em São Paulo, N=140 em Fortaleza e N=82 em Brasília.

### Instrumento

As entrevistas estruturadas, com duração média de 15 minutos, utilizaram um questionário de múltipla escolha cobrindo temas como características sociodemográficas, saúde física e mental, Infecções Sexualmente Transmissíveis (ISTs), vulnerabilidade social, comportamentos de risco, saúde reprodutiva, uso de substâncias, histórico de uso de serviços de saúde e motivação para tratamento. O questionário inclui perguntas fixas para comparabilidade entre séries históricas em São Paulo e outras capitais, adaptadas para cada região por meio de grupos focais com profissionais da Rede de Atenção Psicossocial (RAPS) na CAU. As questões foram validadas por entrevistas cognitivas e um piloto, integrando o treinamento dos entrevistadores.

### Medidas

#### Características sociodemográficas

A seção sociodemográfica do questionário seguiu os padrões estabelecidos pelo Instituto Brasileiro de Geografia e Estatística (IBGE)^24^ e incluía as perguntas sobre: gênero (opções: homem, mulher, trans); idade (numérica, aberta); cor da pele (opções: branca, preta, parda, amarela, indígena); escolaridade (opções: sem escolaridade, ensino fundamental, médio e superior, completos e incompletos); estado civil (opções: solteiro, casado/morando junto, divorciado, viúvo); atividade remunerada (opções: sim, não); rede de suporte (opções: sim, não); e tipos de suporte (opções múltiplas: pessoas e serviços na região da cena, outros serviços, família, amigos).

#### Adaptações realizadas no instrumento

A atualização do instrumento utilizado no levantamento foi fundamental para avaliar a configuração de diferentes cenas de uso. As adaptações ocorreram no tópico referente a uso da rede de saúde e assistência, disponível na CAU, inserindo os serviços Consultório na Rua, em Brasília; e CAPS AD (Rua Dona Leopoldina) — Pop Rua, em Fortaleza. Na seção do uso de substâncias, em Brasília, houve a necessidade de inserção de outras substâncias como "Merla", uso de "Crack no Bombril", e "Balão de Maconha (Maconha + Tabaco). Já na seção diagnósticos de doenças, em Fortaleza, houve a necessidade da inserção de "Hanseníase" e "Hipertensão". Outra alteração realizada foi a inclusão de uma questão para a investigação sobre histórico de trabalho análogo ao escravo na seção "Comportamento e exposição a riscos".

#### Tratamento e análise dos dados

A impossibilidade de usar sistemas informatizados para coleta de dados levou à inserção dos dados em planilhas Excel. O tratamento envolveu limpeza, adaptação, verificação de consistência, codificação e conversão para o *software* STATA SE13. Foram calculados desvios padrão estratificados por capital sem ponderação amostral. Devido ao tamanho reduzido da amostra de pessoas trans em Fortaleza e Brasília, não foi possível segmentar a amostra total por gênero. Os testes de hipóteses χ^2^ de Pearson ou Teste Exato de Fisher foram aplicados conforme a natureza e distribuição dos dados em cada indicador para verificar a homogeneidade entre as três subpopulações (capitais como amostras independentes).

### Aspectos éticos

O protocolo de entrevistas do LECUCA obteve aprovação pelo Comitê de Ética em Pesquisa da UNIFESP com pareceres disponíveis na Plataforma Brasil sob registro números CAAE: 46249121.7.0000.5505 e CAAE: 46249121.7.3001.5553.

Mais informações sobre o LECUCA estão disponíveis no website do estudo: https://lecuca.uniad.org.br/sobre-o-levantamento/sobre-o-lecuca/

## RESULTADOS

### Perfil sociodemográfico

De acordo com a Tabela 1, não foram observadas diferenças relevantes quanto à distribuição de gênero nas três capitais estudadas, com uma predominância de homens com idade média de 37,8 anos de idade nas três capitais (DP=10,5; idade mínima 18 e máxima 77). A cor parda foi a mais prevalente nas três capitais, que também apresentaram índices elevados de frequentadores solteiros e com baixo nível de escolaridade, predominando aqueles que não atingiram o ensino médio. A proporção de indivíduos que nunca estudaram foi significativamente maior em Fortaleza.

**Tabela 1:** Indicadores sociodemográficos e de vulnerabilidade.

Variável		SÃO PAULO (N=357)			FORTALEZA (N=140)			BRASÍLIA (N=82)			TOTAL (N=579)		
		n; %	IC95%		n; %	IC95%		n; %	IC95%		n; %	IC95%	
**Gênero**													
	Homem	262; 73,8	68,96	78,13	95; 68,8	60,57	76,06	58; 72,5	61,56	81,27	415; 72,3	68.61	75.94
	Mulher	80; 22,5	18,47	27,19	39; 28,3	21,33	36,41	21; 26,3	17,67	37,12	140; 24,4	21.08	28.13
	Transgênero	13; 3,7	2,13	6,21	4; 2,9	1,08	7,53	1; 1,3	0,17	8,58	18; 3,1	1.99	4.94
**Idade**													
	18-25	43; 14,0	10,51	18,32	16; 11,6	7,19	18,16	9; 11,0	5,75	19,94	68; 12,9	10.28	16.02
	26-60	253; 82,1	77,44	86,04	121; 87,7	81,01	92,24	67; 81,7	71,67	88,75	441; 83,5	80.10	86.45
	60 ou mais	12; 3,9	2,22	6,75	1; 0,7	0,1	5,04	6; 7,3	3,28	15,51	19; 3,6	2.30	5.58
**Cor** *													
	Branco	90; 25,9	21,52	30,74	13; 10,0	5,87	16,54	13; 15,9	9,36	25,59	116; 20,7	17.55	24.28
	Preto	88; 25,3	20,98	30,14	46; 35,4	27,59	44,05	28; 34,2	24,6	45,18	162; 28,9	25.31	32.83
	Pardo	166; 47,7	42,48	52,97	69; 53,1	44,41	61,56	38; 46,3	35,73	57,3	273; 48,7	44.62	52.90
	Amarelo	2; 0,6	0,14	2,28	0,0			0,0			2; 0,4	0.09	1.42
	Indígena	2; 0,6	0,14	2,28	2; 1,5	0,38	6,01	3; 3,7	1,16	10,91	7; 1,2	0.60	2.60
**Estado Civil**													
	Solteiro	270; 75,8	71,11	80,02	94; 71,2	62,84	78,35	56; 69,1	58,12	78,33	420; 73,8	70.04	77.27
	Separado/divorciado	33; 9,3	6,66	12,77	15; 11,4	6,94	18,07	8; 9,9	4,96	18,7	56; 9,8	7.65	12.58
	Casado/amasiado	44; 12,4	9,32	16,22	20; 15,2	9,95	22,4	10; 12,4	6,71	21,62	74; 13,0	10.48	16.03
	Viúvo	9; 2,5	1,32	4,8	3; 2,3	0,73	6,88	7; 8,6	4,13	17,2	19; 3,3	2.14	5.18
**Nível de escolaridade** *													
	Nunca estudou	6; 1,7	0,76	3,71	10; 7,6	4,13	13,68	3; 3,7	1,16	10,91	19; 3,3	2.14	5.18
	Ensino fundamental	232; 65,2	60,05	69,96	86; 65,6	57,05	73,33	46; 56,1	45,08	66,55	364; 64,0	59.93	67.82
	Ensino médio	103; 28,9	24,44	33,88	28; 21,4	15,13	29,30	25; 30,5	21,39	41,41	156; 27,4	23.90	31.24
	Ensino técnico/faculdade	15; 4,2	2,55	6,88	7; 5,3	2,55	10,85	8; 9,8	4,90	18,48	30; 5,3	3.71	7.45
**Atividade remunerada atual** *		5			1								
	Não	228; 68,7	63,46	73,45	76; 58,9	50,16	67,14	57; 70,4	59,41	79,4	361; 66,6	62.52	70.46
**Fonte de renda**					0								
	Não possui nenhuma renda	188; 53,1	47,88	58,27	73; 56,2	47,44	64,5	42; 51,9	40,89	62,64	303; 53,6	49.49	57.72
	Benefício social	188; 43,2	38,13	48,45	50; 38,5	30,43	47,17	33; 40,7	30,48	51,88	236; 41,8	37.76	45.89
	Renda informal	2; 0,6	0,14	2,24	2; 1,5	0,38	6,01	0,0			4; 0,7	0.27	1.87
	Aposentadoria	5; 1,4	0,59	3,36	2; 1,5	0,38	6,01	4; 4,9	1,84	12,6	11; 1,9	1.08	3.49
	Outra fonte	6; 1,7	0,76	3,73	3; 2,3	0,74	6,98	2; 2,5	0,61	9,52	11; 1,9	1.08	3.49
**Moradia**					0								
	Moradia estável	34; 9,5	6,90	13,08	67; 51,5	42,91	60,08	19; 23,5	15,39	34,05	120; 21,2	17.99	24.73
	Moradia não estável	86; 24,2	19,98	28,89	19; 14,6	9,48	21,86	5; 6,2	2,56	14,15	110; 19,4	16.34	22.87
	Situação de rua*	236; 66,3	61,20	71,03	44; 33,9	26,18	42,47	57; 70,4	59,41	79,4	337; 59,4	55.33	63.41
**Como frequenta a cena de uso**													
	Vive na cena de uso	200; 56,8	51,57	61,92	69; 54,3	45,54	62,86	41; 58,6	46,56	69,64	310; 56,5	52.27	60.57
	Passa os dias e dorme esporadicamente	19; 5,4	3,46	8,32	8; 6,3	3,16	12,17	5; 7,1	2,96	16,26	32; 5,8	4.15	8.13
	Passa os dias mas nunca dorme na cena	101; 28,7	24,19	33,66	26; 20,5	14,28	28,46	21; 30,0	20,30	41,90	148; 27,0	23.40	30.84
	Vai na cena apenas para comprar a droga	32; 9,1	6,49	12,59	24; 18,9	12,95	26,73	3; 4,3	1,36	12,69	59; 10,7	8.41	13.63
**Tempo na cena de uso**					2								
	Menos de um mês	16; 4,6	2,8	7,3	2; 1,6	0,39	6,11	7; 9,1	4,34	18,05	25; 4,5	3.05	6.56
	De 1 mês a 1 ano	55; 15,6	12,18	19,82	18; 14,1	9	21,3	13; 16,9	9,98	27,13	86; 15,4	12.67	18.69
	De 1 a 5 anos	79; 22,4	18,37	27,12	38; 29,7	22,36	38,24	16; 20,8	13,04	31,46	133; 23,9	20.51	27.60
	De 5 a 10 anos	64; 18,2	14,48	22,58	15; 11,7	7,15	18,61	12; 15,6	8,99	25,66	91; 16,3	13.49	19.65
	10 anos ou mais	138; 39,2	34,22	44,42	55; 43,0	34,61	51,75	29; 37,7	27,44	49,11	222; 39,9	35.86	43.99
**Histórico de situação de rua antes de ir para cena de uso** *		121; 35,1	30,20	40,28	92; 23,3	16,56	31,82	26; 35,6	25,36	47,38	175; 32,5	28.69	36.61
**Origem**													
	Instituição de tratamento (Comunidade Terapêutica/hospital)	10; 2,9	1,54	5,25	3; 3,2	1,02	9,56	1; 1,2	0,17	8,47	14; 2,7	1.59	4.47
	Instituição acolhimento (albergue/hotel)	21; 6,0	3,95	9,06	4; 4,3	1,59	10,92	4; 4,9	1,84	12,6	29; 5,5	3.87	7.86
	Outra instituição (recluso/fund., casa)	24; 6,9	4,64	10,07	3; 3,2	1,02	9,56	3; 3,7	1,18	11,03	30; 5,7	4.03	8.08
	Própria casa ou de familiares	255; 73,1	68,15	77,47	71; 75,5	65,72	83,25	49; 60,5	49,34	70,65	375; 71,6	67.54	75.27
**Possui rede de suporte social**		132; 41,0	35,73	46,47	60; 50,0	41,06	58,94	9; 11,1	5,82	20,17	201; 38,4	34.35	42.69
**Fonte de suporte social**		190; 59,0	53,53	64,27	60; 50,0	41,06	58,94	61; 88,9	79,83	94,18	322; 61,6	57.31	65.65
	Serviços no território	37; 16,1	11,87	21,45	6; 7,5	3,37	15,88	11; 15,3	8,58	25,73	54; 14,1	10.98	18.02
	Outros serviços	12; 5,2	2,98	8,99	3; 3,8	1,19	11,17	6; 8,3	3,74	17,54	21; 5,5	3.61	8.29
	Família	149; 64,8	58,36	70,71	52; 65,0	53,8	74,76	49; 68,1	56,28	77,91	250; 65,4	60.52	70.06
	Conhecidos/amigos*	46; 20,0	15,3	25,7	34; 42,5	32,03	53,69	18; 25,0	16,23	36,45	98; 25,6	21.51	30.29

* Indica significância (p<0,05) no teste de homogeneidade entre as capitais (Teste χ^2^ ou Teste Exato de Fisher, dependendo do indicador analisado).

### Indicadores de vulnerabilidade e rede de suporte social

Conforme apresentado na Tabela 1, as capitais foram homogêneas quanto ao índice de frequentadores sem nenhuma fonte de renda, porém diferem quanto à proporção de frequentadores que recebem de algum tipo de programa de transferência de renda (maior prevalência em Brasília) e que referem não realizar nenhuma atividade remunerada (menor índice foi encontrado em Fortaleza). A maioria dos frequentadores das CAUs em São Paulo e Brasília encontram-se em situação de rua, contudo o índice apresenta diferenças significativas em Fortaleza, onde identifica-se alta proporção (51,5%) de frequentadores que referem ter moradia fixa.

As capitais se assemelham no que tange à alta proporção de frequentadores que referem ter vindo de suas casas para a CAU, e pouco mais de um terço dos respondentes em São Paulo e Brasília referiram ter estado em situação de rua antes de frequentarem a CAU — esse índice foi de apenas um quarto da amostra de Fortaleza.

A proporção de novos frequentadores (até um ano) foi semelhante nas três capitais, com São Paulo apresentando mais frequentadores antigos (cinco anos ou mais). Em todas as capitais, mais da metade dos entrevistados vivem na CAU (passam a maior parte do dia e dormem lá). A rede de suporte social variou: em São Paulo e Fortaleza quase metade não possui contatos para emergências, enquanto em Brasília apenas 11,1% relataram essa ausência. Entre os que possuem suporte, a família é a principal fonte em todas as capitais, seguida por conhecidos e amigos.

### Consumo de substâncias e busca por serviços de emergência

De acordo com a Tabela 2, nem todos os frequentadores da CAU referem o consumo de crack. Essa prevalência não foi homogênea entre as capitais, com índices semelhantes (quase 80%) em São Paulo e Fortaleza, porém com menos de um terço dos participantes referindo o uso de crack em Brasília, que também se diferenciou das demais capitais com menores prevalências de uso de cocaína aspirada e maconha. O indicador de consumo de alto risco mensurado através da necessidade de serviços de emergência em decorrência da droga foi mais alto em Brasília, com um terço dos respondentes referindo busca recente por serviços de emergência.

**Tabela 2:** Indicadores de saúde.

VARIÁVEL		SÃO PAULO			FORTALEZA			BRASÍLIA			TOTAL (N=579)		
		n; (%)	IC95%		n; (%)	IC95%		n; (%)	IC95%		n; %	IC95%	
**Consumo de substâncias (uso no último ano)**													
	Crack*	277; 78,9	74,32	82,88	108; 77,7	69,96	83,9	25; 30,9	21,67	41,88	410; 71,8	67.96	75.35
	Cocaína aspirada*	133; 37,9	32,95	43,1	56; 40,3	32,4	48,71	10; 12,2	6,63	21,37	199; 34,8	30.99	38.80
	Maconha	220; 62,2	56,96	67,07	99; 71,2	63,08	78,19	34; 41,5	31,2	52,53	353; 61,4	57.34	65.30
	Álcool	226; 63,7	58,51	68,52	89; 64,0	55,65	71,63	63; 76,8	66,33	84,81	378; 65,6	61.64	69.40
	Solvente inalante	30; 8,7	6,14	12,18	14; 10,1	6,02	16,38	10; 12,2	6,6	21,44	54; 9,5	7.38	12.26
**Uso de serviços de emergência pelo consumo**													
	Não	244; 73,5	68,46	77,98	89; 73,0	64,31	80,15	35; 48,6	37,15	60,22	368; 70,0	65.90	73.74
	Sim, no último ano	62; 18,7	14,83	23,25	12; 9,8	5,64	16,61	24; 33,3	23,3	45,15	98; 18,6	15.52	22.20
	Sim, há mais de um ano	26; 7,8	5,38	11,27	21; 17,2	11,45	25,05	13; 18,1	10,68	28,87	60; 11,4	8.95	14.43
**Histórico de testagem de IST e tuberculose**													
	Tuberculose	205; 61,4	56,02	66,47	70; 53,9	45,17	62,3	41; 50,0	39,19	60,81	316; 57,9	53.68	61.96
	HIV	198; 59,3	53,91	64,44	79; 61,2	52,49	69,32	49; 59,8	48,68	69,92	326; 59,8	55.63	63.86
	Sífilis*	202; 60,7	55,29	65,79	62; 47,7	39,18	56,34	47; 57,3	46,27	67,68	311; 57,1	52.86	61.17
	Hepatite B*	146; 43,8	38,59	49,24	47; 36,2	28,3	44,83	45; 54,9	43,89	65,41	238; 43,7	39.55	47.88
	Hepatite C*	145; 43,4	38,17	48,8	42; 32,3	24,77	40,89	43; 53,1	42,08	63,8	230; 42,2	38.11	46.40

* Indica significância (p<0,05) no teste de homogeneidade entre as capitais (Teste χ^2^ ou Teste Exato de Fisher, dependendo do indicador analisado).

### Histórico de testagem de ISTs e saúde reprodutiva

Menos de 60% da população estudada relata histórico de já ter testado para ISTs (Tabela 2). Apenas Brasília teve pelo menos metade da população da CAU testada para todas as ISTs. Em média, Fortaleza teve a menor taxa de testagem, seguida por São Paulo e Brasília. A tuberculose e o HIV foram as infecções mais testadas, enquanto hepatites B e C foram as menos testadas, ficando abaixo de 50% em São Paulo e Fortaleza.

Sexo desprotegido foi reportado por mais de um terço dos participantes das CAUs (35% na amostra geral), sendo 31,9% em São Paulo, 46,4% em Fortaleza e 28,6% em Brasília. O uso de contraceptivos reversíveis de longa ação (implantes ou "Larc") foi referido apenas em São Paulo, com prevalência de uso em 16,2% das mulheres.

A maioria das mulheres referiu ter tido pelo menos uma gestação (completa ou não). O número médio de gestações na vida ficou entre 3,4 (São Paulo) e 5,0 (Fortaleza), e 3,5 em Brasília. A idade da primeira gestação foi 18 anos, em média, com idade mínima de 10 anos e máxima de 36 anos (as médias por capitais são: São Paulo 18,3 anos; Fortaleza 16,5 anos e Brasília 19,3 anos). Entre as mulheres com histórico de gestação, cerca de 70% relataram histórico de pelo menos um problema em gestações anteriores. As complicações mais reportadas foram aborto (natural ou espontâneo), que chegou a 63,3% de casos em Fortaleza, e prematuridade, alcançando 54,6% em Brasília.

### Histórico de uso da rede de serviços na vida

Conforme Tabela 3, os serviços mais utilizados foram Hospitais Gerais e Unidades Básicas de Saúde (UBS), com mais da metade da população estudada referindo histórico de ter acessado esses equipamentos pelo menos uma vez na vida. O maior acesso à rede de saúde e assistência se destaca em Brasília, onde a maioria (85,4%) refere ter acessado hospitais gerais e mais da metade da amostra frequentou o CAPS-AD. A capital federal também teve altos índices de respondentes que referiram ter acessado grupos de mútua ajuda, como Alcoólicos Anônimos (AA). Houve diferença significativa entre as capitais também quanto ao uso de hospitais psiquiátricos e CAPS-AD, onde Fortaleza apresentou índices muito inferiores às demais capitais, com apenas 8,53% e 9,3% de histórico de atendimento nos dois serviços, respectivamente.

**Tabela 3:** Histórico de uso de serviços da raps (uso na vida).

	SÃO PAULO			FORTALEZA			BRASÍLIA			TOTAL		
	N; %	IC95%		N; %	IC95%		N; %	IC95%		N; %	IC95%	
Hospitais Gerais*	247; 70,6	65,57	75,13	72; 55,8	47,07	64,21	70; 85,4	75,8	91,57	389; 69,4	65.39	73.03
Hospitais psiquiátricos*	72; 20,9	16,89	25,5	11; 8,5	4,76	14,82	17; 20,7	13,2	31,02	100; 18,0	15.00	21.41
UBS*	175; 50,6	45,3	55,84	89; 69,0	60,42	76,43	53; 64,6	53,58	74,32	317; 56,9	52.75	60.98
CAPS	44; 12,9	9,7	16,86	12; 9,3	5,33	15,74	6; 7,3	3,28	15,51	62; 11,2	8.83	14.13
CAPS-AD*	74; 21,6	17,53	26,26	34; 26,4	19,42	34,7	47; 57,3	46,27	67,68	155; 28,0	24.39	31.87
Centro de convivência*	57; 16,7	13,07	21,01	34; 26,4	19,42	34,7	34; 41,5	31,2	52,53	125; 22,6	19.30	26.29
Profissional particular*	6; 1,7	0,71	3,86	5; 3,9	1,61	9,04	13; 15,8	9,36	25,59	24; 4,3	2.92	6.40
Alcoólicos anônimos*	32; 9,4	6,69	12,95	6; 4,6	2,09	10,04	20; 24,4	16,2	34,98	58; 10,5	8.19	
Narcóticos anônimos	50; 14,5	11,18	18,68	6; 4,6	2,09	10,04	15; 18,3	11,25	28,33	71; 12,8	10.26	15.85
Comunidades terapêuticas*	55; 16,1	12,55	20,38	14; 10,8	6,5	17,57	28; 34,1	24,6	45,18	97; 17,5	14.59	20.95
Entidades religiosas	49; 14,3	10,96	18,42	12; 9,3	5,33	15,74	10; 13,4	7,52	22,79	71; 12,8	10.29	15.90

* Indica significância (p<0,05) no teste de homogeneidade entre as capitais (Teste χ^2^ ou Teste Exato de Fisher, dependendo do indicador analisado).

## DISCUSSÃO

A replicação do levantamento para as capitais Fortaleza e Brasília, além de São Paulo, permitiu a compreensão de novas perspectivas quanto ao fenômeno que envolve a formação e consolidação de cenas abertas de uso de crack no Brasil. Os resultados apresentados permitiram compreender os perfis dos frequentadores das cenas de uso estudadas, bem como oferecem reflexões quanto às particularidades de cada território e às necessidades comuns.

Observou-se um paralelo entre todas as CAUs estudadas quanto à existência de um contexto propício para que pessoas que fazem uso de substâncias ilícitas encontrem um ambiente para consumo entre pares, no qual passam, em sua maioria, a morar por um tempo prolongado. O elevado número de pessoas em situação de rua em São Paulo e Brasília é um indício da maneira de relacionar-se com os moradores locais, quando comparado à baixa prevalência em Fortaleza. Diferente das demais capitais, a CAU de Fortaleza integra-se, de uma maneira distinta, com a comunidade local. Uma pesquisa etnográfica na região identifica a presença de mulheres vendendo drogas, evidenciando que a venda do crack está incorporada à "rotina dos afazeres diários"^25^. Embora tenha preenchido os critérios que definem o conceito de CAU do LECUCA, o território em Fortaleza foi considerado "híbrido" e caracterizado pelo uso recluso da droga.

Em São Paulo, a CAU apresenta características únicas em relação à espacialidade do território, ao comportamento e à identidade social de seus frequentadores. Uma das materialidades de identificação com a CAU de São Paulo é o uso dos cachimbos na rua^26^, contrapondo-se ao uso recluso, observado em Fortaleza, e mais discreto, observado em Brasília, onde também se notou uma possível subnotificação de consumo de crack. Combinadas, essas observações levam a especulações sobre diferenças entre as capitais no que se refere ao estigma relacionado ao uso da droga.

Os resultados sugerem uma relação complexa entre situação de rua e uso de drogas: cerca de dois terços dos frequentadores vieram de residências próprias ou de familiares antes de passarem a viver na CAU. Em todas as capitais, a maioria nunca esteve em situação de rua antes de frequentar a CAU, o que contradiz as narrativas que fazem alusão ao uso de crack como decorrência da situação de rua. Esses achados indicam que transtornos de uso de substâncias, ao afetarem a condição econômica e os laços sociais, podem anteceder a situação de rua, ao mesmo tempo que o consumo também pode ser uma adaptação às adversidades enfrentadas nas ruas^27^.

As capitais apresentam altas taxas de sexo desprotegido e problemas em gestações anteriores, com destaque para os altos índices de aborto em Fortaleza e Brasília, indicando a necessidade de iniciativas de planejamento familiar para mulheres em elevada vulnerabilidade que frequentam as cenas de uso. Em São Paulo, esses índices são menores devido à oferta tímida, mas existente, de métodos contraceptivos de longa duração (implantes ou "Larc"). Em Brasília, observa-se o maior índice de atendimentos de emergência relacionados ao uso de drogas, possivelmente por causa da circulação de drogas mais potentes, como variantes da "merla". Esse cenário é compensado pelo maior acesso a serviços de saúde, como UBS e CAPS-AD.

Os índices de acesso aos serviços de rede SUS e SUAS evidenciam a importância da existência de equipamentos nas proximidades da CAU. Apresentando os mais altos índices de acessibilidade para praticamente todos os equipamentos investigados, a CAU em Brasília se caracteriza por ter no seu epicentro um CAPS-AD. Os frequentadores da CAU de São Paulo também desfrutam da proximidade de diferentes equipamentos de saúde e assistência, com ainda maiores índices de acesso ao CAPS Adulto. Isso é evidenciado na alta prevalência do uso da UBS em Fortaleza, sendo esse o único equipamento do SUS disponível no território. Tais achados demonstram a necessidade de políticas públicas que promovam a implementação de estratégias efetivas de prevenção primária e secundária, dentro e fora dos territórios das CAUs.

Os achados demonstram uma perda de janelas de oportunidade para intervenção precoce antes que os indivíduos migrem para as CAUs, denunciando, dessa forma, a urgente demanda por abordagens mais integradas, desde a rede de saúde primária até mesmo na especializada, que não possuem protocolos de rastreamento de abuso de substâncias sob o ponto de vista da prevenção secundária e intervenção precoce.

As diferenças profundas quanto ao perfil e aos tipos de vulnerabilidades demandam uma rede integrada de cuidados, com uma articulação intersetorial eficaz, que permita o fluxo contínuo entre serviços que atuem não apenas nos aspectos relacionados à saúde, mas também que contemplem as necessidades integrais dos indivíduos nessas circunstâncias de vulnerabilidade extrema.

É essencial que a rede de saúde ofereça um cuidado sensível à diversidade de gênero e às necessidades de saúde da mulher, com abordagens tecnicamente qualificadas e acolhedoras. Assim, aproxima-se do princípio da integralidade do SUS, que valoriza direitos humanos e aspectos sociais, priorizando uma trajetória de cuidado focada na ressocialização, além das dimensões técnicas da saúde.

Este estudo apresenta limitações que impactam a interpretação e a generalização dos resultados. A principal delas é a obtenção de amostras representativas de apenas uma CAU por capital, embora existam outras cenas nessas cidades. Apesar de as CAUs escolhidas serem consideradas principais por profissionais da RAPS e pelo estudo exploratório, elas não refletem toda a população de frequentadores dessas capitais, exigindo cautela na generalização dos dados. Possíveis vieses de auto-seleção e de não resposta também devem ser considerados. Além disso, a exclusão de frequentadores com alto grau de intoxicação pode ter gerado um viés, uma vez que esses casos graves ficaram fora da amostra. Embora o questionário seja adaptado para essa população, ainda há potencial para viés de resposta, o que pode afetar a confiabilidade. Acredita-se, no entanto, que o aprimoramento das questões ao longo das edições anteriores em São Paulo e a exclusão dos mais intoxicados possam atenuar esse viés.

O LECUCA oferece subsídios importantes para gestores governamentais e institucionais, com o intuito de catalisar o desenvolvimento de políticas públicas e estratégias de atendimento fundamentadas em dados e evidências. Uma revisão sobre políticas públicas para população em situação de rua no Brasil destaca a importância da ação intersetorial como estratégia para contemplar as múltiplas vulnerabilidades do grupo^28^. De maneira similar, os achados aqui descritos demonstram que as CAUs estudadas compartilham a necessidade de ações integradas, articuladas e sistemáticas que, além de garantir o acesso à saúde e assistência, incluam também serviços complementares. Essas alternativas ampliam as possibilidades de recuperação para indivíduos com transtornos aditivos nas cenas de uso.
